# Response to “Rapid tests for HIV type discrimination in West Africa may perform differently”

**DOI:** 10.7448/IAS.18.1.19380

**Published:** 2015-01-29

**Authors:** Boris K Tchounga, Didier K Ekouevi, Serge P Eholie

**Affiliations:** 1 ISPED, Centre INSERM U897-Epidémiologie-Biostatistique, Université de Bordeaux, Bordeaux, France; 2 Inserm U897, ISPED, Université de Bordeaux, Bordeaux, France; 3 Programme PACCI, Site de Recherche ANRS, Abidjan, Côte d'Ivoire; 4 Département de Santé Publique, Faculté des Sciences de la Santé, Université de Lomé, Lomé, Togo; 5 CHU de Treichville Service des Maladies Infectieuses et Tropicales, Abidjan, Côte d'Ivoire; 6 Département de Dermatologie & Maladies Infectieuses, Université Felix Houphouët Boigny, Abidjan, Côte d'Ivoire

Our study was not designed to evaluate the performances of the two rapid HIV tests, namely Genie II^®^ HIV-1/HIV-2 (BioRad) and SD Bioline^®^ HIV-1/2 3.0 (Standard Diagnostics). Our aim as epidemiologists and clinicians was to clearly make the difference between HIV-1, HIV-2 and dually infected patients, as it is critical for selecting an appropriate treatment strategy. For this purpose, we used the most suitable HIV tests, ImmunoCombII^®^ HIV-1&2 BiSpot (Alere) [1] and an in-house Elisa test [2–4].

Based on the field experience in Guinea-Conakry and Guinea-Bissau [5–7], the authors argue on the need to assess the individual performance of each test used for the initial discrimination of HIV-positive patients. Our team previously conducted in 2004 a field evaluation of rapid HIV serologic tests in Côte d'Ivoire and highlighted the lower accuracy of Genie II for differentiating between HIV-1, HIV-2 and dually reactive patients [4].

In our most recent study, the initial HIV diagnostic tests were reported for 373 patients (68.3% of the overall sample), namely GenieII for 172 (46.1%) and SD Bioline for 79 (21.2%). In Burkina-Faso, 116 samples (50.0%) had the initial HIV test reported; seven (6.0%) of these samples were tested with Genie II and 74 (63.8%) with Bioline. In Côte d'Ivoire, 217 (81.0%) had the initial HIV test reported, among which 145 (66.8%) were tested with Genie II and only four (1.8%) with Bioline. In Mali, this was the case for 40 samples (85.1%), Genie II for 20 samples (50%) and Bioline for one sample (2.5%).

Second, we compared HIV screening results based on these two tests, to the concordant results of a combination of in-house Elisa and ImmunoCombII. Among the 57 samples tested using Bioline, 14 out of 18 initially classified as HIV-2 (77.8%) were confirmed HIV-2 and only six out of 39 initially classified as HIV-1&2 (15.4%) were confirmed HIV-1&2 (kappa=0.19; *p*≤0.001). The final concordant results with in-house Elisa and ImmunoCombII were available for 170 samples initially tested with Genie II. Among the 129 samples initially classified as HIV-2, 121 (93.8%) were confirmed HIV-2 and among the 41 initially classified as HIV-1&2, 16 (39.0%) were confirmed HIV-1&2 (kappa=0.50; *p*≤0.001).

It is clear that these two tests (Genie II and SD Bioline) have different but generally low diagnostic accuracy for HIV discrimination. As we stated in our article, there is a need to systematically retest HIV-1&2 dually reactive patients with more accurate algorithms before treatment initiation.

Finally, we agree with the comment on the use of problematic lots of Bioline tests; however, we did not have access to pharmaceutical data of the AIDS control programme in each country and were therefore unable to provide more information on this question. Nevertheless the participating countries have now updated their national algorithm to introduce more accurate discriminative tests such as Genie III (Côte d'Ivoire,) and ImmunoComII (Mali).
